# O7-6 Global Matrix 3.0 Physical Activity Report Card for Children and Youth: A comparison across Europe

**DOI:** 10.1093/eurpub/ckac094.054

**Published:** 2022-08-29

**Authors:** Anna Chalkley, Tara Coppinger, Elaine Murtagh, Deirdre Harrington, Danielle Johansen, Jan Seghers, Thomas Skovgaard, Karen Milton

**Affiliations:** 1 School of Sport, Exercise and Health Sciences, Loughborough University, Loughborough, United Kingdom; 2 Sport, Leisure & Childhood Studies Department, Cork Institute of Technology, Cork, Ireland; 3 Mary Immaculate College, University of Limerick, Limerick, Ireland; 4 Diabetes Research Centre, University of Leicester, Leicester, United Kingdom; 5 Research and Innovation Centre for Human Movement and Learning, UCL University College & University of Southern Denmark, Odense, Denmark; 6 Physical Activity, Sports & Health Research Group, KU Leuven, Brussels, Belgium; 7 Norwich Medical School, University of East Anglia, Norwich, United Kingdom

**Keywords:** Heath promotion, School, Environment, Community

## Abstract

**Background:**

The Global Matrix of report card grades on physical activity serves as a public health awareness tool by summarising the status of child and youth physical activity prevalence and action. Since schools and the wider community and environment are critical influences on the physical activity levels of children and youth, this research sought to examine the factors considered when assigning these grades across included European countries. Specifically, we sought to: (1) provide a detailed examination of the evidence informing these indicators across participating European Global Matrix 3.0 countries; (2) explore the comparability of the grades for these two indicators across Europe; (3) detail any limitations or issues with the methods used to assign grades; and (4) provide suggestions on how future grading of the indicators could be improved.

**Methods:**

Key documents relating to the European countries involved in the 2018 Global Matrix 3.0 were collated. This inlcuded the long and short forms of the report card for each country as well as the scientific paper. A template was developed and used to capture information on: the grade assigned for each indicator; details of the data used to assign the grade, the source of the data; indication of the quality of the data and any reported challenges or issues in assigning the grade for both the ‘School' and ‘Community and Environment' indicators.

**Results:**

Seventeen of the 20 European Report Card countries (85%) had a grade for schools, and 15 countries (75%) had a grade for community and environment. All countries considered between one and five factors when assigning the grade for these indicators. There were wide disparities in the number and sources of evidence used to assign the grades for both indicators, limiting the comparability of the evidence between different countries.

**Conclusions:**

To enable comparability, the authors recommend moving towards an agreed standardised set of metrics for grading each indicator. Furthermore, it would be useful to develop and share common tools, methods and instruments in order to collect data in a uniform way across countries. Such action will ultimately make the Global Matrix a more robust tool for future use.

